# Solute Carrier Family 1 (*SLC1A1*) Contributes to Susceptibility and Psychopathology Symptoms of Schizophrenia in the Han Chinese Population

**DOI:** 10.3389/fpsyt.2020.559210

**Published:** 2020-09-23

**Authors:** Wenqiang Li, Xi Su, Tengfei Chen, Zhen Li, Yongfeng Yang, Luwen Zhang, Qing Liu, Minglong Shao, Yan Zhang, Minli Ding, Yanli Lu, Hongyan Yu, Xiaoduo Fan, Meng Song, Luxian Lv

**Affiliations:** ^1^ Department of Mental Health, The Second Affiliated Hospital of Xinxiang Medical University, Henan Mental Hospital, Xinxiang, China; ^2^ Henan Key Lab of Biological Psychiatry, International Joint Research Laboratory for Psychiatry and Neuroscience of Henan, Xinxiang Medical University, Xinxiang, China; ^3^ Department of Psychiatry, University of Massachusetts Medical School/UMass Memorial Medical Center, Worcester, MA, United States

**Keywords:** schizophrenia, *SLC1A1*, single-nucleotide polymorphisms, psychopathology symptoms, association

## Abstract

**Objective:**

Schizophrenia (SZ) is a common and complex psychiatric disorder that has a significant genetic component. The glutamate hypothesis describes one possible pathogenesis of SZ. The solute carrier family 1 gene (*SLC1A1*) is one of several genes thought to play a critical role in regulating the glutamatergic system and is strongly implicated in the pathophysiology of SZ. In this study, we identify polymorphisms of the *SLC1A1* gene that may confer susceptibility to SZ in the Han Chinese population.

**Methods:**

We genotyped 36 single-nucleotide polymorphisms (SNPs) using Illumina GoldenGate assays on a BeadStation 500G Genotyping System in 528 paranoid SZ patients and 528 healthy controls. Psychopathology was rated by the Positive and Negative Symptom Scale.

**Results:**

Significant associations were found in genotype and allele frequencies for SNPs rs10815017 (*p* = 0.002, 0.030, respectively) and rs2026828 (*p* = 0.020, 0.005, respectively) between SZ and healthy controls. There were significant associations in genotype frequency at rs6476875 (*p* = 0.020) and rs7024664 (*p* = 0.021) and allele frequency at rs3780412 (*p* = 0.026) and rs10974573 (*p* = 0.047) between SZ and healthy controls. Meanwhile, significant differences were found in genotype frequency at rs10815017 (*p* = 0.015), rs2026828 (*p* = 0.011), and rs3780411 (*p* = 0.040) in males, and rs7021569 in females (*p* = 0.020) between cases and controls when subdivided by gender. Also, significant differences were found in allele frequency at rs2026828 (*p* = 0.003), and rs7021569 (*p* = 0.045) in males, and rs10974619 in females (*p* = 0.044). However, those associations disappeared after Bonferroni’s correction (*p*’s > 0.05). Significant associations were found in the frequencies of four haplotypes (AA, CA, AGA, and GG) between SZ and healthy controls (*χ* ^2^ = 3.974, 7.433, 4.699, 4.526, *p* = 0.046, 0.006, 0.030, 0.033, respectively). There were significant associations between rs7032326 genotypes and PANSS total, positive symptoms, negative symptoms, and general psychopathology in SZ (*p* = 0.002, 0.011, 0.028, 0.008, respectively).

**Conclusion:**

The present study provides further evidence that *SLC1A1* may be not a susceptibility gene for SZ. However, the genetic variations of *SLC1A1* may affect psychopathology symptoms.

## Introduction

Schizophrenia (SZ) is a complex disease with multiple susceptibility genes (1). The pathogenesis of SZ is unknown, and the glutamate hypothesis is one possible suggestion (2). Previously, our studies have revealed susceptibility genes (e.g., SCL1A6) in the glutamate pathway (3). This suggests that research on the glutamate pathway can provide important evidence for the pathogenesis of SZ. At present, a large number of genome-wide association study (GWAS) have revealed that SZ is a complex disease involving multiple genes (4–8). However, there are no consistent results for the important genetic susceptibility genes of SZ. Therefore, identifying SZ susceptibility genes from numerous candidates is an ongoing challenge.

Glutamate is a key primary excitatory neurotransmitter that plays a critical role in synaptic plasticity, neuronal toxicity, neuronal development, and signal transduction in the brain (9), and glutamatergic dysfunction could be involved in the pathogenesis of SZ (2, 10). The glutamatergic dysfunction hypothesis, supported by previous and our recent studies that involved glutamate receptor genes, such as GRIN2A (11), GRIN2B (12), and NRG1 (13, 14), and genes related to glutamatergic transmission, e.g., SCL1A6 (3) and SLC1A3 (15). Thus, further exploration of the genes of the glutamate pathway is important for the research of susceptibility genes for SZ.

The solute carrier family 1 gene (*SLC1A1*), a member of the neuronal high-affinity glutamate transporter family, is located at 9p24.2 and codes for the excitatory amino acid transporter (EAAT) 3 (16), and is expressed throughout the central nervous system, especially in the forebrain (17). Previous studies have reported that *SLC1A1* is associated with risk of SZ (16, 18–20). Expression changes of *SLC1A1* transcripts in SZ have strongly implicated it in SZ pathophysiology (16). Moreover, a 5-generation Palauan family study revealed that an *SLC1A1* mutation and co-segregation correlated with the pathophysiology of SZ (20). Recent GWAS also suggested this gene as an SZ susceptibility gene (21). Some studies have reported that *SLC1A1* SNP rs2228622 (15) and rs7022369 (19) were susceptibility markers involved in the pathogenesis of SZ. Meanwhile, rs16921385 of *SLC1A1* was found to be associated with treatment response to risperidone in SZ (22). Those studies provide more evidence that variation of *SLC1A1* may play a critical role in SZ pathogenesis. However, the findings were inconsistent and scarce (15, 19, 23). Therefore, we further explored the association between *SLC1A1* and SZ in the Chinese Han population.

## Materials and Methods

### Subjects

SZ patients were recruited as inpatients of the Second Affiliated Hospital of Xinxiang Medical University (China) from March of 2005 to December of 2008. The diagnostic criteria of SZ were according to the Diagnostic and Statistical Manual of Mental Disorders-Fourth Edition (DSM-IV). Psychopathology symptoms were measured by Positive and Negative Symptom Scale (PANSS) (24). As in our previous studies (3, 12), family history (FH) was used to explore genetic susceptibility. Inclusion and exclusion criteria of SZ patients and healthy controls were in line with our previous studies (12, 25). Inclusion of SZ: 1) patients with paranoid SZ according to DSM-IV; 2) PANSS ≥ 60; 3) male vs. female = 1:1; 4) Age range from 18 to 55 years old; 5) Han Chinese population. Inclusion of healthy controls: 1) Han Chinese population; 2) male vs. female = 1:1; 3) Age range from 18 to 55 years old. Both SZ and healthy controls were born and lived in the north of Henan Province (China), and unrelated individuals of Chinese Han population. Individuals with other psychiatric disorders, substance dependence, organic brain disease, and severe medical complications were excluded. In the sample collection, the clinical raters had rich experience in administering psychopathological tests and ensure the inter-rater consistency of diagnoses and test results through the training of every 6 months. This study protocol was approved by the Ethics Committee of the Second Affiliated Hospital of Xinxiang Medical University (China). All subjects were informed and signed a written informed consent form.

### SNP Selection

In this study, we used the FASTSNP online service (26) to perform functional analysis, and all 36 SNPs covering the genomic region chr9: 4477575 - 4576808. Meanwhile, those SNPs were a minor allele frequency ≥0.05 and highly ranked risk in the Chinese Beijing population at the HapMap database.

### eQTL Analysis

Further, explore significant SNPs affect the expression level of SLC1A1 gene in brain tissues according to public eQTL databases (BrainSeq Phase 1: http://eqtl.brainseq.org/phase1/eqtl/; BrainSeq Phase 2: http://eqtl.brainseq.org/phase2/eqtl/; Brain xQTL: http://mostafavilab.stat.ubc.ca/xQTLServe/snp_query/).

### Genotyping

Peripheral blood samples were collected from SZ and healthy controls by using evacuated tubes containing EDTA anticoagulant. RelaxGene Blood DNA System (Tiangen Biotech, Beijing, China) was used to extract genomic DNA from white blood cells. The method of genotyping was described in our previous studies that use the Illumina GoldenGate assays on a BeadStation 500G Genotyping System (Illumina, San Diego, CA, USA) (3, 12, 25).

### Statistical Analyses

Statistical analyses were in line with and described in our previous studies (3, 12, 25). G*Power software was used to calculate power (http://www.gpower.hhu.de/). The Haploview V4.1 program was used to assess alleles, genotypes, and haplotype frequency (27). One-way analysis of variance (ANOVA) tests were used for association analyses between PANSS scores and different genotype (SPSS version 25.0, SPSS, Inc. Chicago, IL, USA). Statistical significance was set as *p* < 0.05. Bonferroni correction was used to adjust for multiple testing.

## Results

A total of 1,056 subjects, 528 SZ and 528 healthy controls, were included in this study. There were no significant differences in age or gender between cases and healthy controls (*p* = 0.095, 1.000, respectively) (**Table 1**).

**Table 1:** Demographics of the schizophrenia patients and healthy controls.

Variables	SZ	HC	*p*
N	528	528	
Age (years)	27.32 ± 8.03	27.73 ± 8.01	0.95
Age of Onset (years)	23.47 ± 8.26	NA	
Duration of Illness (years)	6.18 ± 5.91	NA	
Gender (male/female)			1.00
Male	264	264	
Female	264	264	
Family history			
Yes	82	0	
No	446	528	

The genotypes of 36 SNPs were detected in 1056 samples, with a genotyping success rate of 99.79%. There were significant associations in genotype frequency and allele frequency at rs10815017 (*p* = 0.002, 0.030; respectively) and rs2026828 (*p* = 0.020, 0.005, respectively) between cases and healthy controls. Additionally, there were significant associations in genotype frequency at rs6476875 and rs7024664 between cases and healthy controls (*p* = 0.020, 0.021, respectively), and significant associations in allele frequency at rs3780412 and rs10974573 between cases and healthy controls (*p* = 0.026, 0.047, respectively). Meanwhile, there was an association trend in genotype frequency at rs7021569 and rs4742007 between SZ and health controls (*p* = 0.05 for both). Those associations disappeared after Bonferroni’s correction (*p*’s > 0.05). There were no significant associations in genotype frequency or allele frequency at the other 28 SNPs between the two groups (**Table 2**).

**Table 2:** Genotype and allele frequencies of 36 SNPs in the *SLC1A1* gene in SZ patients and healthy controls.

SNP#	dbSNP ID	Allele(D/d)^a^	SZ								HCs								P value	
			N^b^	HWE (p)	Genotype			Allele		MAF	N^b^	HWE (p)	Genotype			Allele		MAF	Genotype	Allele
					DD	Dd	dd	D	d				DD	Dd	dd	D	D			
1	rs10815017	G/A	528	0.021	391	119	18	901	155	0.147	528	0.111	348	168	12	864	192	0.182	**0.002 (0.08)** ^*^	**0.030 (1.00)** ^*^
2	rs2026828	A/G	528	0.240	204	238	86	646	410	0.388	528	0.716	163	257	108	583	473	0.448	**0.020 (0.73)** ^*^	**0.005 (0.22)** ^*^
3	rs6476875	A/G	528	0.001	353	143	32	849	207	0.196	526	0.906	316	184	26	816	236	0.224	**0.020 (0.71)** ^*^	0.111
4	rs7024664	T/A	527	0.000	258	174	95	690	364	0.345	523	0.000	283	132	108	698	348	0.333	**0.021 (0.76)** ^*^	0.540
5	rs7021569	C/G	528	0.877	309	189	30	807	249	0.236	526	0.003	295	180	51	770	282	0.268	0.050	0.088
6	rs4742007	A/G	528	0.041	131	287	110	549	507	0.480	527	0.240	139	250	138	528	526	0.499	0.051	0.384
7	rs3780412	A/G	528	0.832	323	181	24	827	229	0.217	527	0.665	292	198	37	782	272	0.258	0.078	**0.026 (0.94)** ^*^
8	rs10974573	A/C	528	0.364	338	173	17	849	207	0.196	528	0.748	369	146	13	884	172	0.163	0.124	**0.047 (1.00)** ^*^
9	rs7860087	G/C	528	0.555	387	132	9	906	150	0.142	528	0.375	416	103	9	935	121	0.115	0.099	0.059
10	rs2039291	C/A	528	0.529	174	252	102	600	456	0.432	528	0.994	148	263	117	559	497	0.471	0.186	0.073
11	rs2228622	G/A	528	0.858	320	183	25	823	233	0.221	528	0.605	297	195	36	789	267	0.253	0.200	0.082
12	rs10739062	G/C	528	0.359	246	222	60	714	342	0.324	528	0.799	219	240	69	678	378	0.358	0.235	0.098
13	rs10974619	G/A	528	0.522	464	61	3	989	67	0.063	528	0.353	448	75	5	971	85	0.080	0.329	0.130
14	rs12682807	A/C	527	0.805	292	202	33	786	268	0.254	528	0.857	316	184	28	816	240	0.227	0.334	0.147
15	rs2072657	A/C	526	0.107	254	234	38	742	310	0.295	527	0.613	285	202	40	772	282	0.268	0.124	0.166
16	rs16921385	A/G	528	0.177	377	143	8	897	159	0.151	528	0.413	366	144	18	876	180	0.170	0.134	0.213
17	rs10491731	A/C	528	0.687	309	192	27	810	246	0.233	528	0.087	300	186	42	786	270	0.256	0.175	0.224
18	rs3780413	G/C	528	0.089	276	222	30	774	282	0.267	528	0.558	304	190	34	798	258	0.244	0.130	0.231
19	rs188537	C/A	528	0.628	396	124	8	916	140	0.133	528	0.194	410	114	4	934	122	0.116	0.368	0.235
20	rs10814995	A/C	528	0.369	265	212	51	742	314	0.297	528	0.676	242	234	52	718	338	0.320	0.343	0.258
21	rs3087879	G/C	528	0.219	422	97	9	941	115	0.109	526	0.697	405	112	9	922	130	0.124	0.491	0.293
22	rs301432	A/T	528	0.540	327	174	27	828	228	0.216	527	0.427	308	194	25	810	244	0.231	0.421	0.390
23	rs12378107	G/C	506	0.776	396	104	6	896	116	0.115	499	0.101	385	102	12	872	126	0.126	0.345	0.423
24	rs3780411	G/C	528	0.077	131	284	113	546	510	0.483	527	0.632	135	258	134	528	526	0.499	0.213	0.460
25	rs10814991	A/G	528	0.201	152	276	100	580	476	0.451	527	0.292	162	271	94	595	459	0.435	0.760	0.480
26	rs7021409	G/A	528	0.661	267	214	47	748	308	0.292	527	0.643	274	209	44	757	297	0.282	0.884	0.616
27	rs7032326	A/G	528	0.138	213	232	83	658	398	0.377	528	0.893	212	244	72	668	388	0.367	0.581	0.653
28	rs1471786	G/A	528	0.820	152	265	111	569	487	0.461	527	0.807	156	264	107	576	478	0.454	0.939	0.724
29	rs301430	G/A	528	0.211	213	255	60	681	375	0.355	528	0.584	218	238	72	674	382	0.362	0.420	0.751
30	rs6476879	C/A	528	0.284	212	254	62	678	378	0.358	527	0.312	216	251	60	683	371	0.352	0.957	0.775
31	rs10758624	G/A	528	0.247	347	167	14	861	195	0.185	528	0.086	353	150	25	856	200	0.189	0.131	0.780
32	rs10758632	C/G	528	0.437	197	244	87	638	418	0.396	527	0.267	195	241	91	631	423	0.401	0.943	0.797
33	rs3780415	A/G	528	0.671	373	140	15	886	170	0.161	527	0.527	376	136	15	888	166	0.157	0.966	0.827
34	rs7045401	A/C	528	0.277	196	261	71	653	403	0.382	528	0.889	203	250	75	656	400	0.379	0.791	0.893
35	rs301431	C/G	528	0.678	284	209	35	777	279	0.264	527	0.380	281	213	33	775	279	0.265	0.946	0.979
36	rs972519	G/C	528	0.547	452	72	4	976	80	0.076	528	0.547	452	72	4	976	80	0.076	1.000	1.000

SZ, schizophrenia; HCs, healthy controls.

a Major and minor alleles are denoted by D and d, respectively.

b Number of samples with successful genotype.

*p value of Bonferroni correction.

The bold value indicates a value less than 0.05.

When the subjects were divided by sex, we found significant differences in genotype frequency at rs10815017 (*p* = 0.015), rs2026828 (*p* =0.011), and rs3780411 (*p* = 0.040) in males, and rs7021569 in females (*p* = 0.020) between cases and controls. Also, significant differences were found in allele frequency at rs2026828 and rs7021569 in males (*p* = 0.003, 0.045, respectively), and rs10974619 in females (*p* = 0.044) (**Supplementary Table 1**).

We also observed significant differences in genotype frequency at rs10814991 (*p* = 0.015) and rs1471786 (*p* = 0.046) between patients with and without family histories (FH) of SZ. A difference trend was found in genotype frequency at rs3780411 between FH (+) and FH (−) in SZ but was not significant (**Supplementary Table 2**).

**Figure 1:**
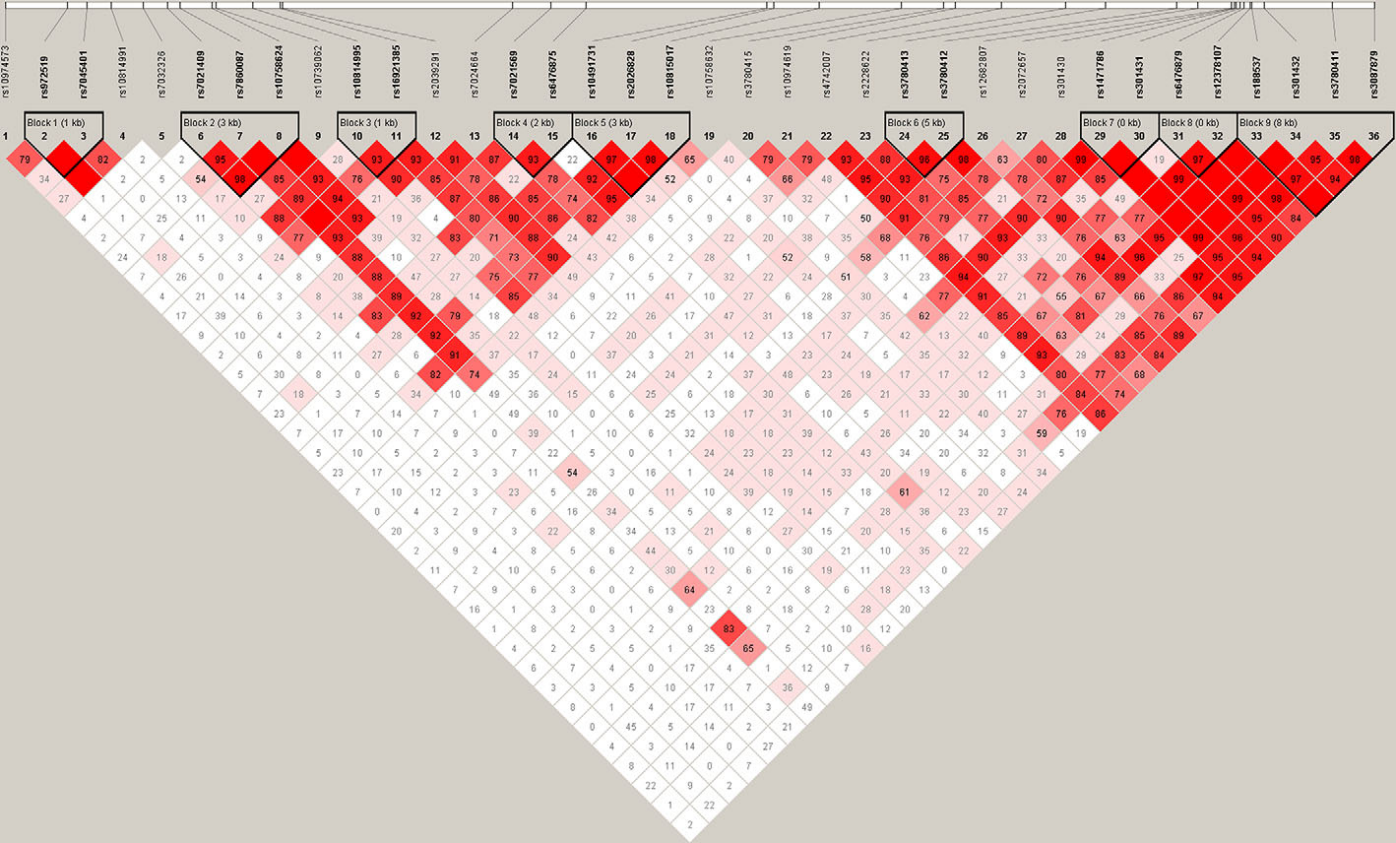
Haplotype block structure of the *SLC1A1* gene in both SZ patients and HCs. The index association SNP is represented by a diamond. The colors of the remaining SNPs (circles) indicate LD with the index SNP based on pairwise r^2^ values from our data.

As shown in **Figure 1**, 9 LD blocks and 32 haplotypes were formed from 36 SNPs. There were significant associations in the frequencies of four haplotypes (AA, CA, AGA, and GG) between SZ and healthy control (*χ* ^2^ = 3.974, 7.433, 4.699, 4.526, *p* = 0.046, 0.006, 0.030, 0.033, respectively) (**Table 3**).

**Table 3:** Associated haplotype frequencies of 36 SNPs in the *SLC1A1* gene between SZ and healthy controls.

Block	Haplotype	Frequencies	SZ Frequencies	HC Frequencies	Chi Square	P Value
1	GA	0.544	0.545	0.543	0.017	0.896
	GC	0.380	0.379	0.382	0.018	0.893
	CA	0.076	0.076	0.076	0.001	1.000
2	GGG	0.523	0.525	0.521	0.029	0.864
	GGA	0.187	0.189	0.184	0.099	0.753
	AGG	0.163	0.171	0.154	1.217	0.270
	ACG	0.123	0.110	0.137	3.443	0.064
3	AA	0.534	0.512	0.556	3.974	**0.046**
	CA	0.306	0.317	0.294	1.361	0.243
	AG	0.157	0.168	0.147	1.681	0.195
4	CA	0.541	0.512	0.571	7.433	**0.006**
	GA	0.249	0.264	0.233	2.688	0.101
	CG	0.207	0.220	0.193	2.326	0.127
5	AAG	0.576	0.545	0.606	7.940	0.005
	CGG	0.240	0.251	0.229	1.405	0.236
	AGA	0.162	0.180	0.145	4.699	**0.030**
	AGG	0.016	0.017	0.014	0.276	0.600
6	GA	0.509	0.500	0.517	0.580	0.447
	CA	0.254	0.241	0.266	1.708	0.191
	GG	0.236	0.255	0.216	4.526	**0.033**
7	AC	0.457	0.453	0.461	0.134	0.714
	GC	0.278	0.282	0.275	0.147	0.702
	GG	0.264	0.265	0.264	0.001	0.981
8	CG	0.525	0.522	0.529	0.108	0.743
	AG	0.354	0.352	0.357	0.050	0.823
	CC	0.120	0.126	0.115	0.692	0.406
9	CAGG	0.502	0.495	0.510	0.477	0.490
	CAGG	0.502	0.495	0.510	0.477	0.490
	CTCG	0.217	0.227	0.207	1.185	0.276
	AACG	0.121	0.112	0.130	1.509	0.219
	CACC	0.112	0.120	0.104	1.241	0.265
	CACG	0.038	0.038	0.037	0.003	0.959

Block 1: rs972519- rs7045401; Block 2: rs7021409-rs7860087-rs10758624; Block 3: rs10814995-rs16921385;

Block 4: rs7021569-rs6476875; Block 5: rs10491731-rs2026828-rs1081507; Block 6: rs3780413-rs3780412;

Block 7: rs1471786-rs301431; Block 8: rs6476879-rs12378107; Block 9: rs188537-rs301432-rs3780411-rs3087879.

The bold value indicates a value less than 0.05.

Further, we make association analysis in six significant SNPs of the *SLC1A1* gene in the PGC samples, including European and East Asian population (**Supplementary Table 3**). We found significant associations at rs10974573 in European population (*p* = 0.008, OR=1.031). Meanwhile, there no significant associations at six SNPs affect the expression level of SLC1A1 gene in main brain tissues, including frontal cortex and hippocampus (*p*’s > 0.05, **Supplementary Table 4** **).**

There were significant associations between rs7032326 genotypes and PANSS total, positive symptoms, negative symptoms, or general psychopathology in SZ (*p* = 0.002, 0.011, 0.028, 0.008, respectively). There were significant associations between rs7860087 genotypes and PANSS total, negative symptoms, or general psychopathology in SZ (*p* = 0.011, 0.015, 0.041, respectively). Genotypes of rs2039291 and rs4742007 were associated with positive symptoms (*p* = 0.029, 0.039, respectively). Genotypes of rs301430 were associated with negative symptoms (*p* = 0.031) (**Table 4**).

**Table 4:** Association analyses between SNPs and sub-scores of PANSS in SZ patients.

SNP	Genotype	N	PANSS Total		Positive Symptoms		Negative Symptoms		General Psychopathology	
			Mean	SD	Mean	SD	Mean	SD	Mean	SD
rs7032326	AA	98	92.86	23.57*	26.17	6.46*	22.20	8.33*	44.48	13.34*
	AG	107	83.62	18.88	23.93	5.80	19.86	6.94	39.82	11.40
	GG	32	97.50	23.35	26.50	7.18	23.09	7.24	47.91	12.94
rs7860087	GG	188	87.74	20.15*	24.86	5.87	20.69	7.07*	42.20	11.96*
	CG	46	96.93	28.01	26.76	8.06	23.74	9.54	46.43	15.00
	CC	3	70.67	5.86	23.33	3.79	19.33	5.51	28.00	5.29
rs2039291	AA	49	90.82	22.71	26.71	6.47*	20.04	7.51	44.06	13.33
	AC	119	88.27	20.48	23.95	5.84	21.45	7.54	42.87	11.97
	CC	69	90.04	24.53	26.30	6.78	21.83	8.00	41.91	13.67
rs4742007	AA	69	86.28	21.11	23.75	6.35*	21.20	7.72	41.32	11.71
	AG	107	92.40	23.25	26.19	6.49	21.85	7.64	44.36	13.60
	GG	61	87.33	20.78	25.13	5.89	20.31	7.67	41.89	12.17
rs301430	AA	28	84.82	15.29	26.25	5.69	18.36	6.91*	40.21	7.98
	AG	106	92.20	24.02	25.33	6.66	22.52	7.76	44.35	14.09
	GG	103	87.56	21.42	24.80	6.22	20.77	7.55	42.00	12.21

^*^p < 0.05, compared with other genotypes; LSD tests, Bonferroni.

## Discussion

We investigated *SLC1A1* mutations associated with the pathogenesis and psychopathology symptoms of SZ in a Han Chinese population. None significant differences were found in genotype and allele frequencies of rs10815017 and rs2026828 between SZ patients and healthy controls after Bonferroni’s correction. Therefore, our study suggests that *SLC1A1* may be not a susceptibility gene for SZ. However, we found that rs7032326 genotypes were associated with psychopathology symptoms of SZ.

Previous studies reported the decreased EAAT3 (encoded product of *SLC1A1*) transcript expression (28), and increased expression of transcripts encoding EAAT1 and EAAT2 (29) in SZ. These findings suggested that the glutamate receptors and related molecules were abnormally expressed in glutamatergic synapses in SZ. In addition, expression of *SLC1A1* was decreased following chronic antipsychotic treatment in animal models. Some studies have also reported that SNP rs2228622 and rs7022369 in the *SLC1A1* gene were susceptibility gene sites for SZ (15, 19). Moreover, the SNP rs16921385, located in an intron of *SLC1A1*, was found to be associated with risperidone treatment response (22, 30). Therefore, *SLC1A1* variation was associated with the pathogeny of SZ. However, there were little studies to explore this association (15, 19, 23) and the mechanism of variation in *SLC1A1* still unknown.

In the present study, there were no significant association with SZ at SNPs rs10815017, rs6476875, rs7024664, and rs10974573. These SNPs are novel gene site findings that have not been reported previously. Further, there had a consistent finding in East Asian population of PGC samples. However, significant association was found at rs10974573 in European population indicated that this SNP may be a susceptibility gene site for SZ. Our study also found that rs3087879, rs301430, rs972519, rs10814991, rs7032326, rs7860087, rs3780415, rs3780413, and rs2072657 were not the susceptibility gene sites for SZ, a result that is consistent with previous reports (15, 19, 23). Meanwhile, we found SNPs rs2026828 and rs3780412 were not susceptibility markers for SZ, which is consistent with previous studies (15, 23). Previous studies reported rs2228622 (15) and rs10814995 (19) of *SLC1A1* were susceptibility markers for SZ in a Japanese population, which is inconsistent with our present finding. However, our finding for rs2228622 is in line with research in the Chinese population (23).

In current, there are only three studied (15, 19, 23) reported SNPs of *SLC1A1* in SZ. Thus, our study provides further evidences for the susceptibility of *SLC1A1* in SZ. As is well known, SZ is highly heterogeneous at genetic and symptomatic levels. Haplotypes of AA, CA, AGA, and GG were also associated with SZ and provided further evidence for the susceptibility of *SLC1A1* in SZ. Compared with previous studies (15, 19, 23), our study has the following innovation and advantages: 1) SZ patient selection: only paranoid SZ patients were included and examined; 2) all subjects were living in the north Henan provinces of China and belonged to the same population group, this ensure the consistency of genetic background; 3) more SNP sites (36 SNPs) were tested than previous studies were including 8 SNPs (15), 19 SNPs (19), and 4 SNPs (23). Therefore, our studies were not only reduced the influence of phenotypic heterogeneity, improved the numbers of SNPs for examination.

SZ is characterized by positive symptoms, negative symptoms, disorganization of thoughts, behaviors, and cognitive deficits. Our previous studies observed the genetic basis for SZ psychopathology symptoms, such as *GRIN2B* was related to cognition deficit symptoms (12), and *CDNF2* was related to negative symptoms (25). However, there are few studies regarding the association between SZ psychopathology symptoms and *SLC1A1* (15, 19, 23). In this study, we found SNP rs7032326 was related to positive symptoms, negative symptoms, and general psychopathology. In addition, another four SNPs (rs7860087, rs2039291, rs4742007, and rs301430) were also related to clinical subtype symptoms. Although these SNPs were not the susceptibility markers for SZ, our finding also provided some evidence for the genetic basis for SZ psychopathology symptoms.

The present study also had some limitations. First, independent samples are needed to verify the finding. Second, the sample size that included PANSS scores was small and insufficient, and should be expanded to further explore the association between genotypes and psychopathology symptoms.

## Conclusion

In conclusion, our study provides further evidence that *SLC1A1* may be not a susceptibility gene for SZ in Chinese Han population. These suggesting the variation of SLC1A1 may be not a genetic mechanism of SZ. However, the genetic variations of *SLC1A1* may affect psychopathology symptoms. Therefore, further studies need to explore other susceptibility genes of SZ.

## Data Availability Statement

The original contributions presented in the study are publicly available. This data can be found here: https://www.oebiotech.com/Article/slc1a1jyyj.html.

## Ethics Statement

The studies involving human participants were reviewed and approved by Ethics Committee of the Second Affiliated Hospital of Xinxiang Medical University (China). The patients/participants provided their written informed consent to participate in this study.

## Author Contributions

Author LL designed the study protocol. Authors TC, YY, YZ, MD, YL, and HY conducted sample selection and data management. Authors XS, ZL, LZ, QL, MLS, and XF undertook the genotyping identify and statistical analysis, and authors WL and MS wrote the first draft of the manuscript. All authors contributed to the article and approved the submitted version.

## Funding

This work was supported in part by the National Natural Science Foundation of China (81671330 and 81971252 to LL), Medical Science and Technology Research Project of Henan Province (2018020373 to HY), National Key Research and Development Program of China (2016YFC1307001), High Scientific and Technological Research Fund of Xinxiang Medical University (2017ZDCG-04 to LL), and the support project for the Disciplinary group of Psychiatry and Neuroscience, Xinxiang Medical University.

## Conflict of Interest

The authors declare that the research was conducted in the absence of any commercial or financial relationships that could be construed as a potential conflict of interest.
