# Supplementary material for: Solute Carrier Family 1 (SLC1A1) Contributes to Susceptibility and Psychopathology Symptoms of Schizophrenia in the Han Chinese Population
Source: Front Psychiatry. 2020 Sep 23;11:559210. doi: 10.3389/fpsyt.2020.559210 (PMC7538510; doi:10.3389/fpsyt.2020.559210)
Supplement: Supplementary file 1 [file DataSheet_1.docx]

**Supplementary Table 1** Genotype and allele frequencies with different gender in the *SLC1A1* gene between SZ patients and health controls

| Gender | SNP# | D/d | Group | N | Genotype | | | HWE(*P*) | *P* | Allele | | *P* | OR (95%CI) |
| --- | --- | --- | --- | --- | --- | --- | --- | --- | --- | --- | --- | --- | --- |
|  |  |  |  |  | DD | Dd | dd |  |  | D | d |  |  |
| Male | rs2026828 | A/G | SZ | 264 | 109(0.413) | 114(0.432) | 41(0.155) | 0.165 | **0.015** | 332(0.629) | 196(0.371) | **0.003** | 1.444(1.129-1.847) |
|  |  |  | HC | 264 | 79(0.299) | 127(0.481) | 58(0.220) |  |  | 285(0.540) | 243(0.460) |  |  |
|  | rs10815017 | G/A | SZ | 264 | 195(0.739) | 59(0.223) | 10(0.038) | 0.839 | **0.011** | 449(0.850) | 79(0.150) | 0.072 | 1.344(0.973-1.857) |
|  |  |  | HC | 264 | 169(0.640) | 89(0.337) | 6(0.023) |  |  | 427(0.809) | 101(0.191) |  |  |
|  | rs3780411 | G/C | SZ | 264 | 67(0.253) | 143(0.542) | 54(0.205) | 0.732 | **0.040** | 277(0.525) | 251(0.475) | 0.268 | 1.146(0.900-1.459) |
|  |  |  | HC | 264 | 71(0.269) | 117(0.443) | 76(0.288) |  |  | 259(0.491) | 269(0.509) |  |  |
|  | rs7021569 | C/G | SZ | 264 | 166(0.629) | 81(0.307) | 17(0.064) | 0.025 | 0.155 | 413(0.782) | 115(0.218) | **0.045** | 1.334(1.006-1.769) |
|  |  |  | HC | 264 | 145(0.549) | 95(0.360) | 24(0.091) |  |  | 385(0.729) | 143(0.271) |  |  |
| Female | rs7021569 | C/G | SZ | 264 | 143(0.542) | 108(0.409) | 13(0.049) | 0.299 | **0.020** | 394(0.746) | 134(0.254) | 0.671 | 1.062(0.806-1.399) |
|  |  |  | HC | 262 | 150(0.573) | 85(0.324) | 27(0.103) |  |  | 385(0.735) | 139(0.265) |  |  |
|  | rs10974619 | G/A | SZ | 264 | 237(0.898) | 24(0.091) | 3 (0.011) | 0.040 | 0.079 | 498(0.943) | 30(0.057) | **0.044** | 1.622(1.009-2.608) |
|  |  |  | HC | 264 | 220(0.833) | 41(0.157) | 3 (0.011) |  |  | 481(0.911) | 47(0.089) |  |  |

Note: SZ, schizophrenia; HC, healthy control; The bold value indicates a value less than 0.05.

**Supplementary Table 2** Association between genotype and FH in the SLC1A1 gene with SZ

| SNP# | Genotype | FH+(Frequency) | FH-(Frequency) | P value |
| --- | --- | --- | --- | --- |
| rs10814991 | AA | 38 (38.8%) | 96 (25.3%) |  |
|  | AG | 40 (40.8%) | 212 (55.9%) | 0.015 |
|  | GG | 20 (20.4%) | 71 (18.7%) |  |
| rs1471786 | GG | 28 (28.6%) | 113 (29.8%) |  |
|  | AG | 58 (59.2%) | 182 (48%) | 0.046 |
|  | AA | 12 (12.2%) | 84 (22.2%) |  |
| rs3780411 | GG | 15 (15.3%) | 99 (26.1%) |  |
|  | CG | 59 (60.2%) | 200 (52.8%) | 0.069 |
|  | CC | 24 (24.5%) | 80 (21.1%) |  |

Note: FH, family history.

**Supplementary Table 3** Association analysis in six SNPs of the SLC1A1 gene in the PGC samples.

| SNP# | Ethnicity | Case | Control | OR | SE | *P*-value |
| --- | --- | --- | --- | --- | --- | --- |
| rs10815017 | European | 40675 | 64643 | 0.980 | 0.011 | 0.057 |
|  | East Asian | 14583 | 17261 | 0.991 | 0.022 | 0.676 |
| rs2026828 | European | 40675 | 64643 | 0.999 | 0.001 | 0.895 |
|  | East Asian | 14583 | 17261 | 1.019 | 0.017 | 0.260 |
| rs6476875 | European | 40675 | 64643 | 0.985 | 0.010 | 0.128 |
|  | East Asian | 14583 | 17261 | 1.027 | 0.020 | 0.194 |
| rs7024664 | European | 40675 | 64643 | 0.980 | 0.012 | 0.100 |
|  | East Asian | 14583 | 17261 | 1.006 | 0.017 | 0.745 |
| rs3780412 | European | 40675 | 64643 | 1.004 | 0.010 | 0.703 |
|  | East Asian | 14583 | 17261 | 0.983 | 0.019 | 0.373 |
| rs10974573 | European | 40675 | 64643 | 1.031 | 0.012 | **0.008** |
|  | East Asian | 14391 | 17214 | 1.009 | 0.022 | 0.679 |

OR, odds ratio; SE, standard error; The bold value indicates a value less than 0.05.

**Supplementary Table 4** SNPs affect the expression level of SLC1A1 gene in main brain tissues

| Variant ID | SNP# | Tissue | Effect Size | t | *P*-Value |
| --- | --- | --- | --- | --- | --- |
| chr9_4546594_C_T_b38 | rs10815017 | Frontal Cortex | -0.016 | -0.29 | 0.77 |
|  |  | Hippocampus | -0.051 | -0.89 | 0.38 |
| chr9_4543307_A_G_b38 | rs2026828 | Frontal Cortex | -0.038 | -0.69 | 0.49 |
|  |  | Hippocampus | -0.1 | -1.8 | 0.08 |
| chr9_4529671_T_C_b38 | rs6476875 | Frontal Cortex | -0.0011 | -0.02 | 0.98 |
|  |  | Hippocampus | -0.045 | -0.79 | 0.43 |
| chr9_4524371_A_T_b38 | rs7024664 | Frontal Cortex | 0.039 | 0.54 | 0.59 |
|  |  | Hippocampus | -0.046 | -0.56 | 0.58 |
| chr9_4572480_T_C_b38 | rs3780412 | Frontal Cortex | -0.024 | -0.45 | 0.65 |
|  |  | Hippocampus | -0.021 | -0.37 | 0.71 |
| chr9_4487575_T_G_b38 | rs10974573 | Frontal Cortex | -0.0036 | -0.057 | 0.95 |
|  |  | Hippocampus | -0.083 | -1.1 | 0.26 |
